# When helping hurts: validating a measure of compulsive helping and exploring potential correlates

**DOI:** 10.3389/fpsyg.2025.1504413

**Published:** 2025-02-26

**Authors:** Katey Workman, Laura M. Padilla-Walker, Peter J. Reschke, Adam A. Rogers

**Affiliations:** 1 Department of Psychological Sciences, University of California, Merced, Merced, CA, United States; 2 School of Family Life, Brigham Young University, Provo, UT, United States

**Keywords:** helping, prosocial, anxiety, empathic concern, self-regulation

## Abstract

**Introduction:**

This cross-sectional study proposes and validates a new measure of compulsive helping: helping which harms the helper.

**Methods:**

Emerging Adults (*N* = 438; Mage = 20.29, SD = 1.04, 51.71% Female) reported on compulsive helping. Confirmatory factor analysis was used to assess construct validity, while bivariate correlations were used to assess convergent and discriminant validity.

**Results:**

Confirmatory factor analysis suggested convergent validity with all 10 items loading onto a single factor, with factor loadings above 0.44. Model fit was acceptable. Convergent validity was demonstrated such that compulsive helping was positively correlated with prosocial behavior and anxiety. Discriminant validity was demonstrated such that compulsive helping was negatively associated with self-regulation.

**Conclusion:**

This new concept and measure of compulsive helping is a first step toward defining the limits of the adaptiveness of prosocial behavior. Though not frequent, it appears helping which is harmful is not uncommon. Future research should employ qualitative means and consider the multidimensionality of prosocial behavior.

## Introduction

The adaptiveness of prosocial behavior is largely agreed upon (Padilla-Walker and Carlo, 2015), yet some have called for the limits of its adaptiveness to be defined (Oakley et al., 2012; Eisenberg and Spinrad, 2015). There is theoretical and limited empirical evidence for the costliness of prosociality in the extreme (Niemiec, 2019; Oakley et al., 2012), but to date there is no validated tool dedicated to its measurement. Thus, the present study seeks to propose and validate a new measure for extreme prosociality, herein termed compulsive helping. In the sections that follow we use extant theoretical and empirical work to define compulsive helping, distinguish it from related constructs, and investigate potential correlates.

### Define and distinguish construct

Compulsive helping is conceptualized in this study as a powerful impulsive to help, enacted in a repetitive or excessive way which harms individual functioning. Simply put, it is helping which harms the helper.

Helping is a subtype of prosociality (behaviors intended to help others; Eisenberg et al., 2015; Carlo and Padilla-Walker, 2020). Another prosocial subtype is altruism, thus our examination of compulsive helping as an extreme form of prosociality is preceded by the study of pathological altruism (Oakley et al., 2012). Though related, the two terms differ in essential ways. *Pathological altruism* implies severity, diagnosability, and pureness of motive that we contend are not necessarily relevant to all forms of prosocial behavior, therefore inadvertently excluding behaviors of relevance. The selection of the alternative term *compulsive helping* is intended to capture more quotidian, non-clinical, and variously motivated actions intended to benefit others.

What both constructs have in common, however, is a dearth of empirical attention. The Oxford Manual on Pathological Altruism (Oakley et al., 2012), while theoretically rich, lacks a validated measure for the construct. Compulsive helping likewise lacks a construct-specific validated measure and the term only receives verbatim mention in the PROMIS questionnaire and its derivations (Christo et al., 2003; and use of those measures seldom reference the item except to specify its value as an avenue for future research). This empirical gap is one we strive to address.

Despite an empirical paucity, there is theoretical basis for helping that is harmful. Optimal usage framework posits that traits or strengths optimally used are adaptive, but when used at extremely low or high levels become problematic (Freidlin et al., 2017). For instance, generosity is a strength when optimally used, but under-usage (i.e., never lending money to a friend in need) could jeopardize a friendship, and over-usage (i.e., giving too frequently) may leave the giver without funds for basic needs. Thus helping, may likewise appear adaptive when used optimally, and problematic when over-used (e.g., compulsive helping). This framework justifies the examination of magnitude in addition to presence or absence of a trait (e.g., generosity) or behavior (e.g., helping) as important to full understanding of a given construct.

### Identify and justify correlates

The validity of the measure we propose will be determined in part by comparison to anticipated correlates. Based on theory and empirical precedent, we will inspect four: prosocial behavior, empathic concern, self-regulation, and anxiety.

Prosocial behavior is the broader construct of which helping is a subtype (Schroeder and Graziano, 2015). In accordance with optimal usage theoretical framework previously discussed, prosocial behavior is anticipated to positively correlate with compulsive helping as the two comprise the respective optimal- and extreme-iterations of helping. What is more, when the intended recipient (i.e., target) of prosocial behavior is considered, differences in both motivation and outcome are often observed (Padilla-Walker and Fraser, 2014). Thus, associations are expected to differ between the proposed measure of compulsive helping and prosocial behavior toward family, friends, and strangers.

In contrast, empathic concern (affective state of being concerned or compassionate; Davis, 1983) at normative levels tends to correspond to normative helping, it follows, therefore, that extreme empathic concern (e.g., empathic distress; Smith and Rose, 2011) should correspond with extreme (i.e., compulsive) helping. Thus, a relationship with normative empathic concern is likely to be either weakly or uncorrelated with compulsive helping.

Self-regulation (individual control that occurs in response to environmental exigencies; Novak and Clayton, 2001) is anticipated to inversely correlate with compulsive helping in accordance with the theoretical framework of addiction. An addiction is characterized by an inability to control impulses (4th ed.; DSM–IV; American Psychiatric Association, 1994). It may be that an unregulated impulse to help others is a useful way to conceptualize compulsive helping.

Finally, empirical work informs the final correlate of interest: anxiety (the apprehension, tension, or uneasiness that stems from the anticipation of danger, which may be internal or external; 4th ed.; DSM–IV; American Psychiatric Association, 1980). Social anxiety may motivate helping (Culotta and Goldstein, 2008), especially in the case of non-familiar others, as an attempt to preempt negative appraisal or garner acceptance. We have argued that pathology and compulsion are distinct. And yet, since anxiety has been empirically linked with the development of pathology (Adams et al., 2019), as well as with prosocial behavior (Memmott-Elison et al., 2020), it seems worthwhile to assess whether it plays a role in our conceptualization of compulsive helping.

### Emerging adulthood and compulsive helping

The current sample is comprised of emerging adults. This developmental period is germane to the present question for its neurobiological and social particularities. Specifically, it is at this stage that mental health disorders often emerge (De Girolamo et al., 2012). Also, still-developing self-regulatory capacities may facilitate a degree of compulsiveness (Blakemore, 2008; Steinberg, 2010). Socially, greater autonomy may motivate the increase of helping observed at this stage (Carlo and Padilla-Walker, 2020), to the extent that autonomous helping is associated with greater satisfaction (Weinstein and Ryan, 2010). Yet, the tensions which accompany transitioning to living on one’s own with its ensuing personal boundary re-negotiation, comparative relational instability, and self-focus (Arnett, 2015), may prime helping which is excessive or maladaptive (i.e., compulsive helping) in search of acceptance and stability. Not intended for clinical use, the proposed measure may nevertheless assist future research on emerging adulthood to understand the nuances of behavior that is otherwise adaptive. In turn, this knowledge may assist implicated emerging adults to establish health patterns on the path to flourishing in adulthood (Nelson and Padilla-Walker, 2013).

### Current study

To begin defining the limits of the adaptability of prosocial behavior, the current study seeks to propose and perform initial tests of validity for a new measure of compulsive helping. To test convergent validity, compulsive helping will be correlated with general prosocial behavior (toward family, friends, and strangers), empathic concern, and anxiety. It is anticipated that greater prosocial behavior will correspond with greater compulsive helping with slight variation by target (family, friends, strangers) of behavior. Empathic concern is anticipated to be either weakly positively correlated or uncorrelated with compulsive helping. Anxiety is anticipated to be positively correlated with compulsive helping. Finally, discriminant validity will be examined through testing associations with self-regulation. It is anticipated they will be inversely correlated and demonstrate slight variation by dimension (emotional, cognitive, behavioral) though their examination will be exploratory.

## Methods

### Participants

Participants for this study were drawn from wave 10, collected in 2017, of the Flourishing Families Project (see Padilla-Walker et al., 2025). Participants were recruited using a multi-method approach used to mirror the socio-economic and racial-ethnic stratification of the area, then randomly selected, and compensated upon completion. Participants (N = 438) were aged 18–23 (M*age* = 20.29, SD = 1.04, 51.71% Female). Sixty-nine percent of participants identified as European American (12.8% African American, 11.18% Multi-ethnic, 4.14% Asian American, 1.45% Hispanic, 0.83% were identified as “Other”; see Table 1 for demographic information).

**Table 1:** Flourishing families demographic statistics table (*N* = 439).

Variables	M	SD	Range
Age	20.29	1.04	18–23
Compulsive Helping	0.35	0.34	0–2
Self-Regulation	3.69	0.43	1-4
Emotional	4.20	0.64	1-4
Cognitive	3.28	0.6	1-4
Behavioral	3.43	0.63	1-4
Anxiety	1.23	0.69	0–3
Empathic Concern	3.94	0.62	1-5
General PB	4.17	0.53	1-5
PB Family	4.34	0.68	1-5
PB Friend	4.60	0.55	1-5
PB Stranger	3.58	0.79	1-5
Race/ethnicity			
African American	12.84%		
Asian American	4.14%		
European American	69.57%		
Hispanic	1.45%		
Multi-ethnic	11.18%		
Other	0.83%		
Gender			
Female	51.71%		
Male	47.84%		
Other	0.46%		

### Measures

The compulsive helping measure was adapted from a video game addiction scale, taken from a national study of pathological video game use (Gentile, 2009; Original Cronbach’s alpha = 0.78). *Helping* replaced the term *video game* on items such as “I have needed friends or family to give me extra money because I have spent too much money *helping others*” (see Table 2 for items). One item was dropped once deemed irrelevant in a helping context, and another replaced the word “homework” with “important tasks” for age appropriateness. Regarding the scale, respondents were given 3 options: no, sometimes, yes, later valued at 0, 0.5, and 1, respectively.

**Table 2:** Confirmatory factor analysis and response frequency for compulsive helping measure.

		Response							
		No		Sometimes		Yes		Alpha if item deleted	Factor loading
Item	Description	*n*	%	*n*	%	*n*	%		(SE)
1	Over time, I have been spending much more time thinking about helping others, learning about new ways to help others, or planning the next opportunity to help.	243	55.48	139	31.74	56	12.79	0.7961	0.44 (0.04)
2	I need to spend more and more time and/or money on helping others in order to feel the same amount of excitement	350	79.91	75	17.12	13	2.97	0.7844	0.52 (0.04)
3	I have tried to help others less often or for shorter periods of time, but am unsuccessful	347	79.22	73	16.67	18	4.11	0.783	0.56 (0.04)
4	I help others as a way of escaping from problems or bad feelings	243	58.41	150	34.25	45	10.27	0.7854	0.53 (0.04)
5	I become restless or irritable when I avoid helping others	285	65.22	125	28.6	27	6.18	0.786	0.51 (0.04)
6	I have lied to family or friends about how much I help others	367	83.79	59	13.47	12	2.74	0.7793	0.6 (0.04)
7	I sometimes skip household chores in order to spend more time helping others	315	71.92	110	25.11	13	2.97	0.7774	0.61 (0.04)
8	I sometimes skip doing important tasks in order to spend more time helping others	271	61.87	138	31.51	29	6.62	0.7741	0.62 (0.04)
9	I have done poorly on assignments because I spend too much time helping others	324	73.97	90	20.55	24	5.48	0.7773	0.61 (0.04)
10	I have needed friends or family to give me extra money because I have spent too much money helping others	366	83.56	59	13.47	13	2.97	0.7932	0.44 (0.04)
Total								0.8010	

After confirmatory factor analyses estimated construct validity, individual mean scores were calculated for compulsive helping and used in subsequent analyses. Cronbach’s alpha for all items was 0.80.

Prosocial behavior was measured using 15 items based on the Inventory of Strengths (Peterson and Seligman, 2004; Original Cronbach’s alpha = 0.70). The measure assessed prosocial behavior directed toward others/strangers (5 items, a modified version of the Peterson and Seligman original measure), friends (5 items), and family (5 items). Respondents answered on a 5-point Likert-type scale, ranging from 1 (*not like me at all*) to 5 (*very much like me*) in terms of how much they disagreed or agreed with statements about themselves. Sample statements included, “I help people I do not know, even if it is not easy for me.” Higher scores indicate greater levels of kindness and generosity toward strangers, family, and friends. A mean score was calculated. Cronbach’s alphas were found to be 0.89 (general), 0.90 (family), 0.88 (friend), and 0.82 (strangers) for the current sample.

Empathic concern was assessed using a 7-item self-report measure from the Interpersonal Reactivity Index (Davis, 1983; Original Cronbach’s alpha = 0.72). The Likert-type response scale ranged from 1 (*strongly disagree*) to 5 (*strongly agree*) and higher scores indicate greater empathic concern. Sample items included, “When I see someone being taken advantage of, I feel kind of protective towards them.” A mean score was calculated. The Cronbach’s alpha coefficient was found to be 0.80 for the current sample.

Anxiety was assessed using a 6-item generalized anxiety disorder subscale (Spence, 1998; Original Cronbach’s alpha = 0.73). Participants responded using a 4-point Likert scale ranging from 0 (*never*) to 3 (*always*) with higher scores reflecting greater levels of anxiety. Sample items included, “I worry a lot about things,” A mean score was calculated. Cronbach’s alpha was 0.89.

Self-regulation was assessed using a modified 13-item measure (Novak and Clayton, 2001; Original Cronbach’s alphas for the emotion, cognitive, and behavioral subscales were 0.95, 0.96, and 0.94, respectively). Responses ranged from 1 (*never true*) to 4 (*always true*). Sample items included, “I get distracted by little things” (reversed). Higher scores represent greater ability to regulate negative emotion, behavior and to reach goals. A mean score was calculated. Cronbach’s alpha coefficients were found to be 0.76 (full scale), 0.81 (emotional subscale), 0.72 (cognitive subscale), and 0.74 (behavioral subscale) for this research sample.

### Analytic strategy

**Figure 1:**
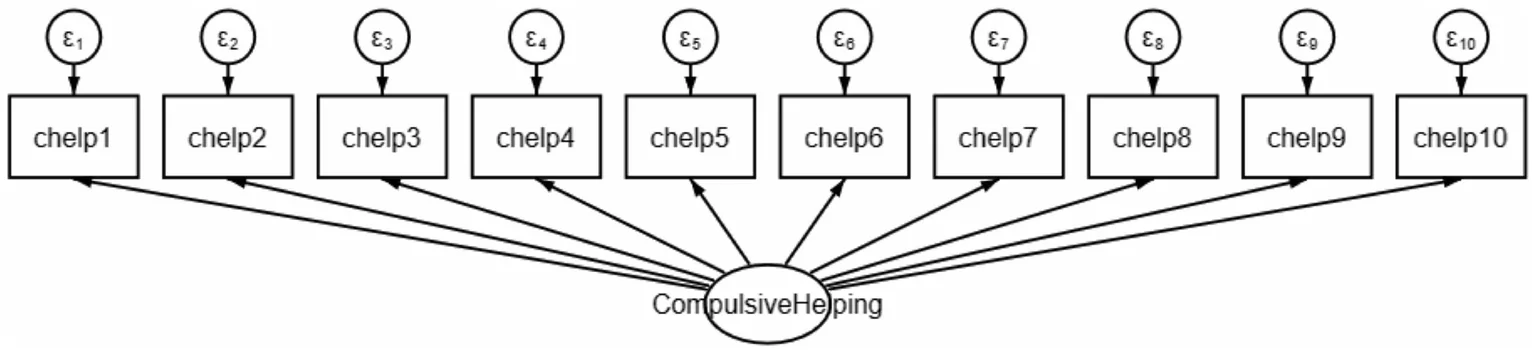
Confirmation factor analysis (CFA) for compulsive helping.

Data were screened for missingness and to verify that model assumptions were satisfied. Construct validity was assessed using confirmatory factor analysis (CFA—see Figure 1). Variance was fixed to 1. Factor loadings above 0.40 (Tabachnick and Fidel, 1996) would indicate validity. Model Fit indices included chi-squared, comparative fit index (CFI), Tucker-Lewis index (TLI) and root mean squared error of approximation (RMSEA). Acceptable fit would be indicated with values of >0.90 (CFI/TLI), 0.05–0.08 (RMSEA) (Kline, 2016). Convergent and discriminant validity were assessed using bivariate correlations.

## Results

Data revealed that across the full sample missing data ranged from 3.78–12.75%. After generating a binary variable of compulsive helping (0 = missing, 1 = non-missing) and estimating a logistic regression, none of the primary study variables (anxiety, self-regulation, empathic concern, or general prosocial behavior) appeared to significantly predict missingness. Missing data was accounted for using maximum likelihood estimation.

### Descriptive statistics

Descriptive results can be seen in Table 1. Reported compulsive helping was low (*M* = 0.35, SD =0.34), while general prosocial behavior was reported at high levels (*M* = 4.17, SD =0.53). Unsurprisingly, prosocial behavior was high toward friends (*M* = 4.59, SD =0.55), and family (*M* = 4.34, SD =0.68), and moderate toward strangers (*M* = 3.58, SD =0.79). Likewise, levels of empathic concern were reportedly moderate (*M* = 3.94, SD =0.62), as were levels of self-regulation (*M* = 3.69, SD =0.43). Performing descriptive analyses of the subtypes of self-regulation indicated that emotional regulation was fairly high (*M* = 4.20, SD =0.64), while behavioral (*M* = 3.43, SD =0.63), and cognitive self-regulation (*M* = 3.28, SD =0.60) were moderate.

### Construct validity

Confirmatory factor analysis (CFA) was estimated with Stata SEM using Maximum Likelihood with Missing Values estimator. All items loaded on a single factor with standardized factor loadings above 0.44, indicating construct validity (Tabachnick and Fidel, 1996). Table 2 demonstrates the factor loadings as well as the frequency of responses per item. Model fit was acceptable, *X*^2^(35) = 88.87, *p* < 0.001, CFI: 0.94; TLI: 0.92, RMSEA: 0.06 (Kline, 2016).

### Convergent validity

Correlational analyses (see Table 3) revealed that general prosocial behavior was positively associated with compulsive helping (*p* = 0.0069) with associations differing as a function of target (family *p* = 0.7644; friends *p* = 0.3357; strangers *p* = 0.000). Anxiety was positively associated with compulsive helping (*p* = 0.000). Empathic concern was unrelated to compulsive helping (*p* = 0.2119).

**Table 3:** Correlations of variables of interest (*N* = 462) with MLMV.

	1.	2.	3.	4.	5.	6.	7.	8.	9.	10.
1. Compulsive Helping	-									
2. Anxiety	0.21***	-								
3. Empathic Concern	0.06	0.14**	-							
4. Prosocial Behavior General	0.13**	0.08	0.56***	-						
5. PB Family	0.01	−0.04	0.39***	0.78***	-					
6. PB Friends	0.05	0.10*	0.42***	0.78***	0.50***	-				
7. PB Strangers	0.22***	0.13**	0.51***	0.81***	0.37***	0.45***	-			
8. Self-Regulation	−0.18***	−0.45***	0.07	0.14**	0.14**	0.10*	0.09	-		
9. SR Emotional	−0.15**	−0.40***	−0.03	0.04	0.02	0.04	0.04	0.72***	-	
10. SR Behavioral	−0.13**	−0.35***	0.06	0.05	0.07	0.01	0.03	0.77***	0.25***	-
11. SR Cognitive	−0.06	−0.08	0.20***	0.28***	0.26***	0.23***	0.17**	0.47***	0.04	0.23***

*p < 0.05, **p < 0.01, ***p < 0.001.

### Discriminant validity

Correlational analyses indicated a negative association between self-regulation and compulsive helping (r = −0.18, *p* = 0.0002), indicating that higher self-regulation was associated with less compulsive helping. When considering subtypes, emotional (*p* = 0.0013) and behavioral (*p* = 0.0079) self-regulation were significantly negatively associated, while cognitive self-regulation (*p* = 0.1823) evinced no significant association with compulsive helping.

## Discussion

The limits of the adaptability of prosocial behavior require definition (Eisenberg and Spinrad, 2015). Compulsive helping provides a possible conceptualization in the form of helping that is harmful. This concept is especially important to explore during emerging adulthood because the developmental context may facilitate both its development and detection (Steinberg, 2010). Insufficient measurement tools currently exist to test this concept. Therefore, the goal of the present study was to propose and validate a measure of compulsive helping.

### Validated measure of compulsive helping

Construct Validity was established using confirmatory factor analyses, suggesting the measure may be of use for future studies. The proportion of respondents who answered “sometimes” and “yes” to compulsive helping items seems to suggest that the tendency, though infrequent by our calculations, may not be uncommon, thus meriting more attention than the topic presently enjoys (Eisenberg et al., 2015).

Convergent validity was established by the positive correlation between compulsive helping and general prosocial behavior (specifically with strangers), anxiety, and demonstrating no relationship with empathic concern. It may be that those willing to disregard personal comfort to enact comparatively high-cost behavior such as helping a stranger (Padilla-Walker and Fraser, 2014) may be likewise suppressive of personal or social signals to stop helping when it goes too far. Anxiety, though tenuously connected with helping generally (Memmott-Elison et al., 2020), may especially contribute when helping is performed at high levels or in high-cost scenarios. Furthermore, social anxiety may take the form of people pleasing, manifested here as helping others (Culotta and Goldstein, 2008). The optimal usage framework (Niemiec, 2019) anticipates the lack of relationship between compulsive helping (extreme) and empathic concern (normative) and offers the possibility that extreme empathy (e.g., empathic distress; Smith and Rose, 2011) may be the extreme iteration of empathy which merits future research as a correlate.

Discriminant validity was established as hypothesized: a negative correlation between compulsive helping and self-regulation generally, including emotional and behavioral dimensions specifically. The original measure from which this proposed compulsive helping measure was adapted was categorized in the DSM-IV as an impulse control disorder (4th ed.; DSM–IV; American Psychiatric Association, 1994). Moral emotions (e.g., shame; Sheikh and Janoff-Bulman, 2010) unregulated may motivate excessive helping. Externalizing behaviors are rooted in under-regulation (Memmott-Elison et al., 2020) meaning the present findings support extant literature. Future research could explore how strengthening emotional and behavioral regulation earlier in adolescence might be protective against later compulsive prosocial behaviors to establish healthy patterns early in development.

### Limitations and future directions

Limitations of the current study include racial and socio-economic homogeneity, cross-sectionality, limited developmental scope, and lack of sociometric expertise. Helping differs across countries and cultures (Carlo and Padilla-Walker, 2020; Jensen, 2011), and patterns may manifest differently in collectivistic and individualistic contexts (Green et al., 2005). The lack of racial and socioeconomic diversity in the sample limits the generalizability of the present findings. Longitudinal data would enable the examination of predictors over time and help to establish directionality of effects. Emerging adulthood, though a helpful starting place, cannot provide insight into relevant features of compulsive helping during other developmental stages. Finally, content experts were consulted in the development of the proposed scale, but additional consultation of those with sociometric expertise would add to its rigor.

Potential avenues for future research include examining gender effects, targets, types and motivations of compulsive behaviors, distinguishing between similar constructs, and performing qualitative research.

Consistently, females help more than males (Eisenberg and Mussen, 1989) despite similar levels of helping during infancy (Eisenberg et al., 2015). Also, females report suffering more frequently from internalizing symptoms including anxiety and personal distress (Broidy and Agnew, 1997; Bromet et al., 2011). Combined, these factors affirm the importance of examining gender in future research on compulsive helping.

Prosocial behavior is multidimensional, with different correlates based on the target, type, and motivation of the behavior (Carlo and Padilla-Walker, 2020). While some acknowledgement of multidimensionality was considered herein, more detailed analyses will likely prove illuminating. Helping which harms the target or intended recipient (as opposed to the helper) might also merit a compulsive designation and yet evince differential correlates (Carlo and Padilla-Walker, 2020) to what was herein considered. Consideration of specific types of helping (e.g., serving, donating, defending, etc.) might reveal that certain types of helping are more prone to compulsivity than others, or that it is instead individual traits which predict compulsion regardless of type. Motivation behind behavior was operationally neglected in this initial study but will likely prove an illuminating avenue of future research, as such a nuanced construct as compulsive helping might emerge from complex (i.e., primary and secondary) motivations (Martin and Olson, 2015).

Finally, future research should employ qualitative methods (e.g., focus groups, individual interviews, observational techniques, etc.; Kahlke, 2014) to understand more precisely the form compulsive helping takes in any given developmental stage. While quantitative methods allow for breadth of inquiry, qualitative methods enable depth and specificity that could greatly benefit the seedling field of compulsive helping (Shakouri and Nazari, 2014).

### Conclusions and implications

Despite the limitations in the present study, and in recognition of the enormous amount of clarifying future research called for, it is believed that the present measurement validation of compulsive helping adds to the prosocial literature in being among the first empirical attempts to define the limits of the adaptiveness of prosociality. Implications of this study on moral psychology broadly include the acknowledgement that helping, prosociality, or moral behavior can be maladaptive. This study does not attempt to definitively designate what makes a behavior adaptive versus maladaptive, only to illustrate that the magnitude of moral action (e.g., helping) in addition to its presence or absence may have relevance for individual adjustment.

Future research is required to parse out the nuances of the construct including directionality, gender differences, correlates, and distinguishing between similar constructs. In so doing, a more holistic understanding of prosociality and the limits of its adaptability can potentially contribute to the establishment of healthy patterns of helping in emerging adulthood and beyond.

## Data Availability

The raw data supporting the conclusions of this article will be made available by the authors, without undue reservation.
