# Correction: When helping hurts: validating a measure of compulsive helping and exploring potential correlates

**DOI:** 10.3389/fpsyg.2026.1941195

**Published:** 2026-08-28

**Authors:** Katey Workman, Laura M. Padilla-Walker, Peter J. Reschke, Adam A. Rogers

**Affiliations:** 1 Department of Psychological Sciences, University of California, Merced, Merced, CA, United States; 2 School of Family Life, Brigham Young University, Provo, UT, United States

**Keywords:** helping, prosocial, anxiety, empathic concern, self-regulation

There was a mistake in Table 1 as published. Typos for range values contained dates (1-Apr, 1-May) instead of response category numerical ranges (1–4, 1–5). The corrected Table 1 appears below.

**Table 1:** Flourishing families demographic statistics table (*N* = 438).

Variables	*M*	SD	Range
Age	20.29	1.04	18–23
Compulsive helping	0.35	0.34	0–2
Self-regulation	3.69	0.43	1–4
Emotional	4.20	0.64	1–4
Cognitive	3.28	0.60	1–4
Behavioral	3.43	0.63	1–4
Anxiety	1.23	0.69	0–3
Empathic concern	3.94	0.62	1–5
General PB	4.17	0.53	1–5
PB family	4.34	0.68	1–5
PB friend	4.60	0.55	1–5
PB stranger	3.58	0.79	1–5
Race/ethnicity			
African American	12.84%		
Asian American	4.14%		
European American	69.57%		
Hispanic	1.45%		
Multi-ethnic	11.18%		
Other	0.83%		
Gender			
Female	51.71%		
Male	47.84%		
Other	0.46%		

There was a mistake in the caption of Table 1 as published. The title for Table 1 reads “Flourishing Families sample demographic statistics table (*N* = 439).” Additionally, the “Race/Ethnicity” sub-title reads (*N* = 483). These were vestiges of prior iterations of the table and should be adjusted simply to reflect final sample size (*N* = 438) and only in the title of the table.

The corrected caption of Table 1 appears below.

The original version of this article has been updated.

